# National utilization of minimally invasive and open liver resections

**DOI:** 10.1007/s00464-026-12678-9

**Published:** 2026-04-20

**Authors:** Vikram S. Pothuri, Katelin B. Nickel, Abdurrahman Abdelzaher, Faiz Gani, Ryan C. Fields, Karen E. Joynt Maddox, Anne M. Butler, Chet W. Hammill

**Affiliations:** 1 Department of Surgery, Washington University School of Medicine, St. Louis, MO USA; 2 Division of Infectious Diseases, Department of Medicine, Washington University School of Medicine, St. Louis, MO USA; 3 Cardiovascular Division, Department of Medicine, Washington University School of Medicine, St. Louis, MO USA; 4 Center for Advancing Health Services, Policy & Economics Research, Washington University School of Medicine and Institute for Public Health, St. Louis, MO USA; 5 Center for Advance Surgery-East, The Oregon Clinic, Portland, OR USA

**Keywords:** Minimally invasive, Laparoscopic, Robotic-assisted, Liver

## Abstract

**Introduction:**

Although minimally invasive (MIS) liver resections are associated with improved outcomes, uptake remains slow.

**Methods:**

Using data from the Healthcare Cost and Utilization Project (HCUP) National Inpatient Sample (NIS), adults who underwent elective liver resections (2016–2020) were identified. Approach was classified as MIS (laparoscopic or robotic-assisted) or open. Annual utilization was characterized by approach and association between approach and patient and hospital variables was estimated.

**Results:**

Among 63,280 adult liver resections, 22.5% were MIS. MIS resections increased from 21.3% in 2016 to 22.5% in 2020. Laparoscopy alone decreased from 18.4 to 14.9%, while robotic-assistance increased from 2.9 to 7.6%. In multivariable analysis, patients were less likely to undergo MIS resection if they were in a county with < 250,000 people (vs. > 250,000, OR [95% CI], 0.82 [0.73–0.93]). Patients were more likely to undergo a MIS resection if they had a benign indication (vs. malignancy, OR [95% CI], 2.9 [2.49–3.38]), or were undergoing a partial hepatectomy (vs. lobectomy, OR, [95% CI], 2.62 [2.24–3.07]).

**Conclusion:**

MIS liver resection increased between 2016 and 2020, driven by an increase in robotic-assisted surgery and despite a reduction in laparoscopic surgery. Rurality, indication, and resection type were contributors to a MIS vs. open approach.

**Supplementary Information:**

The online version contains supplementary material available at 10.1007/s00464-026-12678-9.

With the introduction of laparoscopic liver resection in the 1990s [1], and more recently robotic-assisted liver resections, the available technical approaches for complex hepatic procedures have diversified. Evidence suggests that minimally invasive (MIS) techniques (laparoscopic and robotic-assisted) offer post-operative benefits with equivalent oncologic outcomes compared to open liver resection. The advantages of MIS, particularly laparoscopic, over open liver resections include reduced blood loss, fewer complications, shorter hospital stays, and decreased morbidity [2–5].

Recent randomized clinical trials have demonstrated no difference in survival outcomes between open and laparoscopic liver resections [6], despite reservations regarding surgical margin clearance in the setting of malignancy. Moreover, the implementation of structured training programs has reduced the burden of technical demands and learning curves associated with advanced surgical techniques [7]. Despite these developments, the widespread national adoption of MIS in liver resections in the United States has been slow [8]. Nationally, the vast majority of liver resections continue to be open surgery, despite evidence in support of post-operative benefits and equivalent oncologic outcomes for minimally invasive approaches [6, 8]. However, prior studies are limited by outdated data, a focus on patients undergoing liver resections only for malignancies, and a lack of inclusion of robotic-assisted resections [8, 9].

To fill this gap, a national cohort study was performed to characterize the utilization of open and MIS liver resections from 2016 to 2020. Additionally, this study sought to estimate the association between resection approach and demographic, clinical, and hospital characteristics. Finally, this study sought to estimate the association between resection approach and post-operative outcomes, accounting for demographic, clinical and hospital characteristics.

## Materials and methods

### Data source

This study used data from the Healthcare Cost and Utilization Project (HCUP) National Inpatient Sample (NIS), which is maintained by the Agency for Healthcare Research and Quality (AHRQ) [10]. The NIS is the largest publicly available all-payer inpatient healthcare database, enabling national estimates of inpatient utilization, access, quality, and outcomes. It contains a sample of roughly 7 million hospital stays each year, which consists of a 20% sample of hospital discharges in the United States. The NIS provides a weight variable to project to roughly 35 million hospitalizations nationally [11, 12]. It includes over 100 variables ranging from patient demographics (e.g. age, sex, race/ethnicity, insurance status), clinical characteristics (e.g. diagnoses, procedures, complications, in-hospital mortality), and hospital characteristics (size, region, teaching status).This study was determined to not involve activities subject to Institutional Review Board oversight as HCUP data is anonymized and there were no attempts to identify any subjects.

### Study population

**Fig. 1:**
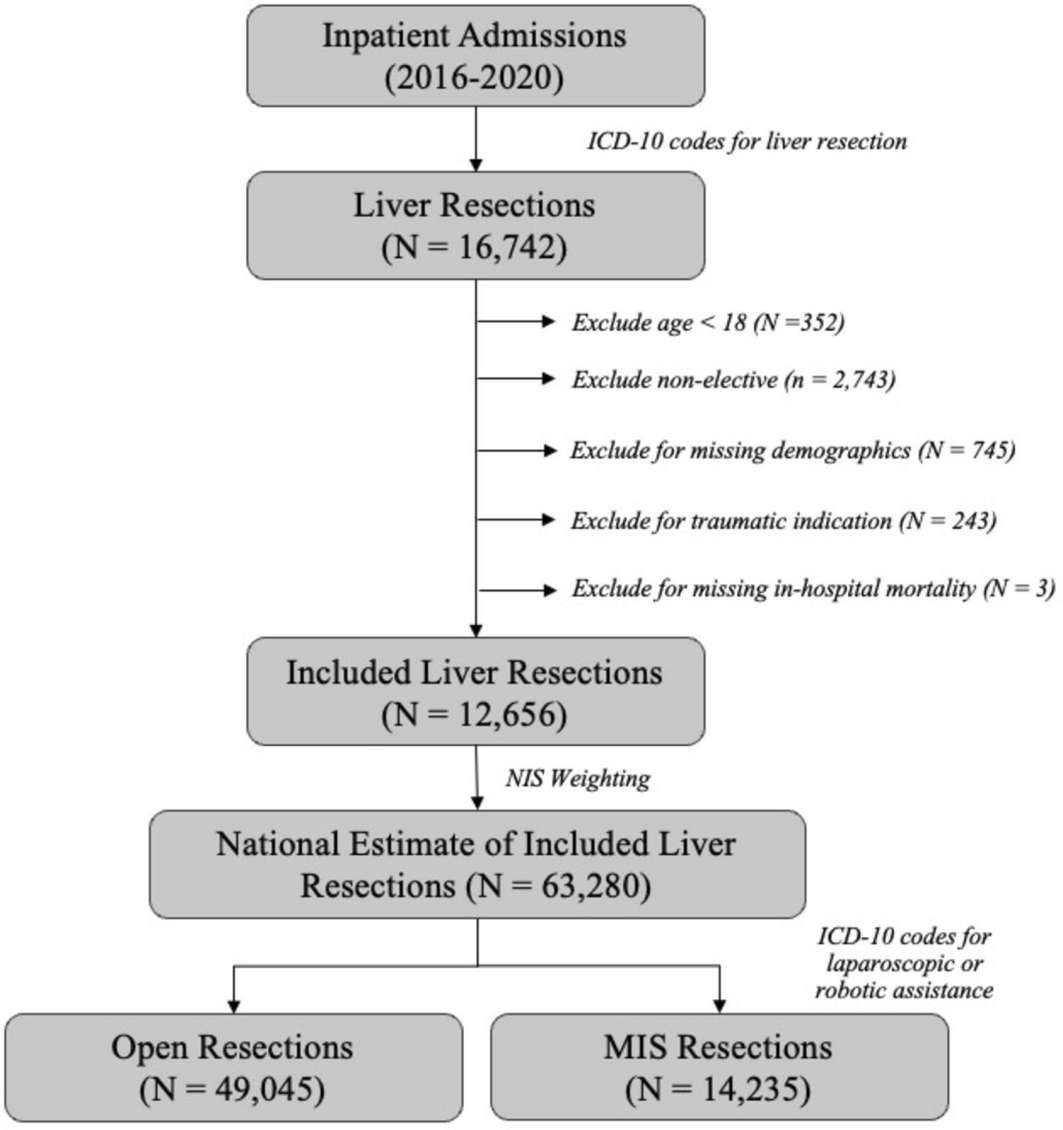
Flow diagram for study population. Liver resections were identified in the NIS data over a 5 year period (2016–2020). Patients were excluded due to age < 18 (*N* = 352), non-elective resections (*N* = 2743), missing demographic variables (*N* = 745), traumatic indication (*N* = 243), or missing in-hospital mortality (*N* = 3). 12,656 relevant liver resections were present in the NIS sample, which translated to 63,280 relevant liver resections using NIS weighting

All liver resections from January 1, 2016 to December 31, 2020 were identified using relevant ICD-10 procedure codes (resection of right lobe [0FT10ZZ, 0FT14Z], resection of left lobe [0FT20ZZ, 0FT24ZZ], excision of liver [0FB00ZZ, 0FB04ZZ], excision of right lobe [0FB10ZZ, 0FB14ZZ], excision of left lobe [0FB20ZZ, 0FB24ZZ]). Resection of right lobe or left lobe were classified as lobectomies and excisions of liver, right lobe, or left lobe were classified as partial hepatectomies. ICD-10 procedure codes include a descriptor that outlines the surgical approach of the procedure. As a result, resection approach was classified as laparoscopic if the ICD-10 code had a percutaneous endoscopic approach descriptor (0FT14Z, 0FT24ZZ, 0FB04ZZ, 0FB14ZZ, 0FB24ZZ). Resections were classified as robotic-assisted if they were classified as laparoscopic based on the aforementioned criteria and an additional code for robotic-assisted procedure of trunk region (8E0W4CZ) was recorded on the same day as the index liver resection. Resection approach was classified as open if the ICD-10 code had an open approach descriptor (0FT10Z, 0FT20ZZ, 0FB00ZZ, 0FB10ZZ, 0FB20ZZ). Total liver resections (0FT00ZZ, 0FT04ZZ) were not included. Resections were excluded based on patient age less than 18 years (*N* = 352), non-elective resections (*N* = 2743), missing demographic variables (*N* = 745), traumatic indication (*N* = 243), or missing in-hospital mortality (*N* = 3) (Fig. 1).

### Outcome definitions

Post-operative complications were identified using ICD-10 code-based definitions that have been previously validated with administrative data [13]. Complications occurred during the index admission and were classified into seven categories: infectious (including pneumonia, urinary tract infection, sepsis, and other infections), wound, end organ dysfunction (including cardiac, renal, respiratory, and global), thromboembolic, accidental laceration or puncture, transfusion, and other (Supplemental File: ICD-10 codes). The end organ dysfunction classification did not include liver failure or any related liver-specific complications, thus capturing overall post-operative sequelae related to the surgery and not post-operative liver dysfunction related to the liver resection itself. In-hospital mortality and length of stay were obtained through the “DIED” and “LOS” variables in the NIS, and are defined as death during hospital admission and number of days between admission date and discharge date respectively.

### Covariates

Several covariates were ascertained during the hospitalization. Indications for liver resections were determined based on ICD-10 codes and categorized as primary malignancy, secondary malignancy, neuroendocrine tumor, benign, laceration, multiple, or other based on prior publications (Supplemental file: ICD-10 codes) [9]. Neuroendocrine tumors were separated from other secondary malignancies as the surgical decision-making for these tumors differs from other malignancies with metastases to the liver. Non-anatomic resections and debulking procedures may be more likely, which may affect decision making regarding minimally invasive vs. open approaches and therefore warrants analysis as a distinct category. Comorbidities were identified using an established ICD-10 code list [14]; the number of comorbidities was subsequently analyzed as a continuous variable. Demographic variables included age, sex, race, insurance status, rurality, and median income by zip code. Hospital level-variables (bed size, location/teaching status, region) were obtained from the associated hospital-level files and merged with patient-level data using hospital identifiers.

## Statistical analysis

Given that the NIS data represent a 20% sample of hospital discharges in the United States, these data were expanded to a national estimate using the svydesign function with NIS_STRATUM as strata and DSCWT as weights. Individual- and hospital-level characteristics were summarized by resection approach. Annual proportion of utilization of each resection approach was plotted over the study period (2016–2020). The Joinpoint Trend Analysis Software (version 5.1.0) developed by the National Cancer Institute was used to model trends and calculate annual percentage change and 95% confidence intervals (CIs) [15]. A multivariable logistic regression model was used to examine factors associated with resection approach. In addition, separate multivariable logistic regression models were used to examine the association between resection approach and each post-operative complication, accounting for demographic factors, clinical factors, and hospital characteristics. Predetermined sub-group analysis was performed with the MIS group to examine differences in utilization and post-operative outcomes between laparoscopic and robotic-assisted approaches. All analyses were performed using R (v 4.2.2).

## Results

### Patient and hospital characteristics

Among 16,742 total liver resections identified during the 5-year study period (2016–2020), 12,656 liver resections were eligible for study. After using NIS stratification and discharge weights to project from a nationally representative sample to a national estimate, the final cohort was comprised of 63,280 liver resections, including 77.5% (*N* = 49,045) open resections and 22.5% (*N* = 14,235) MIS (laparoscopic and robotic-assisted) resections (Fig. 1). Table 1 presents the demographic, clinical, and hospital characteristics, overall and by resection approach. In the overall population, the mean age was 59.3 years and 53.3% of patients were female. Compared with open resection recipients, MIS resection recipients were less likely to be living in rural areas (18.5% vs. 22.4%) or in ZIP codes with median income below the national average (44.1% vs. 46.7%). In addition, MIS resection recipients were less likely than open resection recipients to have an indication of primary malignancy (22.9% vs. 24.6%), secondary malignancy (29.4% vs. 44.5%), neuroendocrine tumor (0.8% vs. 1.5%) and multiple indications (1.8% vs. 3.6%). MIS resection recipients were more likely than open resection recipients to have a benign indication (24.0% vs. 7.4%). MIS resection recipients had a greater proportion of partial hepatectomies (91.0% vs. 78.4%) and lower proportion of lobectomies (8.6% vs. 18.6%) compared to open resection recipients. MIS recipients had a lower mean number of Elixhauser comorbidities than open resection recipients (2.68 vs 3.07). Compared with open resections, MIS resections were less often performed at large hospitals (71.7% vs. 76.7%) or urban teaching hospitals (92.3% vs. 94.2%).

**Table 1:** Demographic, clinical, and hospital characteristics of patients in study population by resection approach

	Overall	Open	Minimally invasive	Standardized mean difference
	*N* = 63,280*	*N* = 49,045	*N* = 14,235	
Patient demographics				
Age, mean (standard deviation)	59.3 (13.9)	59.3 (13.8)	59.4 (14.2)	0.01
Sex				0.12
Male	29,530 (46.7)	23,560 (48.0)	5970 (41.9)	
Female	33,750 (53.3)	25,485 (52.0)	8265 (58.1)	
Race and ethnicity				0.05
White	43,840 (69.3)	34,205 (69.7)	9635 (67.7)	
Black	6685 (10.6)	5145 (10.5)	1540 (10.8)	
Hispanic	6285 (9.9)	4770 (9.7)	1515 (10.6)	
Asian or Pacific Islander	3640 (5.8)	2795 (5.7)	845 (5.9)	
Native American	230 (0.4)	175 (0.4)	55 (0.4)	
Other	2600 (4.1)	1955 (4.0)	645 (4.5)	
Insurance				0.05
Private insurance	29,580 (46.8)	22,880 (46.7)	6700 (47.2)	
Medicare	24,365 (38.6)	18,885 (38.6)	5480 (38.6)	
Medicaid	6110 (9.7)	4785 (9.8)	1325 (9.3)	
Self-pay	1205 (1.9)	970 (2.0)	235 (1.7)	
No charge	195 (0.3)	170 (0.3)	25 (0.2)	
Other	1720 (2.7)	1280 (2.6)	440 (3.1)	
Rurality				0.10
Greater than or equal to 250,000	50,200 (78.5)	38,060 (77.6)	11,600 (82.5)	
Less than 250,000	13,620(21.5)	10,985 (22.4)	2635(18.5)	
Income by Zip Code				0.05
Above median	34,085 (53.9)	26,125 (53.3)	7960 (55.9)	
Below median	29,195 (46.1)	22,920 (46.7)	6275 (44.1)	
Patient clinical characteristics				
Indication				0.52
Primary malignancy	15,310 (24.2)	12,050 (24.6)	3260 (22.9)	
Secondary malignancy	26,000 (41.1)	21,810 (44.5)	4190 (29.4)	
Neuroendocrine tumor	830 (1.3)	720 (1.5)	110 (0.8)	
Benign	7020 (11.1)	3610 (7.4)	3410 (24.0)	
Multiple	2005 (3.2)	1745 (3.6)	260 (1.8)	
Other	12,115 (19.1)	9110 (18.6)	3005 (21.1)	
Surgery type				0.37
Lobectomy	10,350 (16.4)	9125 (18.6)	1225 (8.6)	
Lobectomy and partial hepatectomy	1520 (2.4)	1460 (3.0)	60 (0.4)	
Partial hepatectomy	51,410 (81.2)	38,460 (78.4)	12,950 (91.0)	
Number of elixhauser comorbidities, mean (standard deviation)	2.99 (1.78)	3.07 (1.80)	2.68 (1.69)	0.22
Hospital characteristics				
Hospital bed size				0.12
Small	4225 (6.7)	3185 (6.5)	1040 (7.3)	
Medium	11,250 (17.8)	8265 (16.9)	2985 (21.0)	
Large	47,805 (75.5)	37,595 (76.7)	10,210 (71.7)	
Hospital location/teaching status				0.08
Rural	510 (0.8)	360 (0.7)	150 (1.1)	
Urban non-teaching	3380 (5.3)	2435 (5.0)	945 (6.6)	
Urban teaching	59,390 (93.9)	46,250 (94.3)	13,140 (92.3)	
Hospital region				0.07
Northeast	15,035 (23.8)	11,525 (23.5)	3510 (24.7)	
Midwest	13,635 (21.5)	10,855 (22.1)	2780 (19.5)	
South	21,440 (33.9)	16,610 (33.9)	4830 (33.9)	
West	13,170 (20.8)	10,055 (20.5)	3115 (21.9)	

*Percentages presented in parentheses, unless indicated otherwise

### Trends in utilization of open and MIS liver resections

**Fig. 2:**
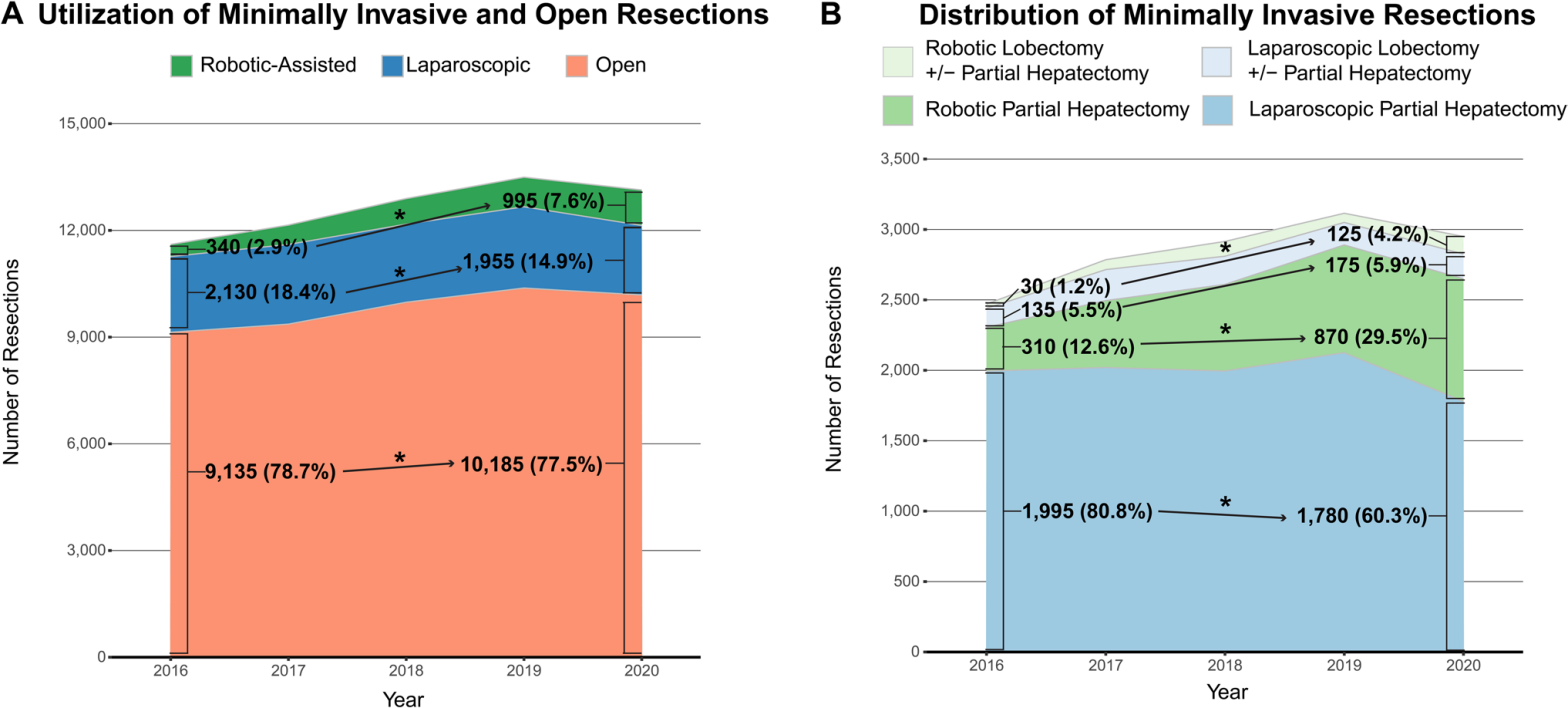
Utilization and breakdown of minimally invasive liver resections. **A** Annual number and percentage of open, laparoscopic and robotic liver resections from 2016–2020. **B** Annual number of and percentage of minimally invasive resections broken down by laparoscopic vs. robotic-assisted and by surgery type (lobectomy ± partial hepatectomy vs. partial hepatectomy)

Over the course of the study period, MIS, including both laparoscopic and robotic-assisted, resections increased from 21.3% (*N* = 2470) of all resections in 2016, to 22.5% (*N* = 2950) of all resections in 2020 (average annual percent change (AAPC) 1.15%, 95% Confidence Interval (CI) [− 3.01 to 5.46%]), while open resection declined from 78.7% (*N* = 9135) in 2016 to 77.5% (10,185) in 2020 (AAPC − 0.32%, 95% CI [− 1.64 to 1.00%]) (Table 2). Despite the overall increase in MIS resections, laparoscopic resections decreased from 18.4% (*N* = 2130) in 2016 to 14.9% (*N* = 1955) in 2020 (AAPC − 4.92%, 95% CI [− 9.78 to 0.10%]). As a result, the increase in MIS resections was driven by growth in robotic-assisted liver resections, which increased from 2.9% (*N* = 340) in 2016 to 7.6% (*N* = 995) in 2020 (AAPC 24.8%, 95% CI [7.83 to 44.1%]) (Fig. 2A). The shift from laparoscopic to robotic-assisted was most pronounced in partial hepatectomies. Among MIS resections, robotic-assisted partial hepatectomies increased from 12.6% (*N* = 310) in 2016 to 29.5% (*N* = 870) in 2020 (AAPC 23.0%, 95% CI [12.6 to 34.4%]), while laparoscopic partial hepatectomies decreased from 80.8% (*N* = 1995) in 2016 to 60.3% (*N* = 1780) in 2020 (AAPC − 6.24%, 95% CI [− 8.41 to − 4.13%]). This trend was not as stark with lobectomy ± partial hepatectomy (2016 vs 2020; laparoscopy alone: 5.5% vs 5.9%, AAPC − 2.63%, 95% CI [− 27.5 to 30.6%]; robotic-assisted: 1.2% vs 4.2%, AAPC 26.0%, 95% CI [− 23.3 to 105.1%]) (Fig. 2B).

**Table 2:** Average annual percent change (AAPC) of resection approaches over study period

Resection approach/type	Average annual percent change (95% confidence interval)
Open	− 0.32% [− 1.64 to 1.00%]
MIS	1.15% [− 3.01 to 5.46%]
Laparoscopic	− 4.92% [− 9.78 to 0.10%]
Partial hepatectomies	− 6.24% [− 8.41 to − 4.13%]
Lobectomy ± partial hepatectomy	2.63% [− 27.5 to 30.6%];
Robotic-assisted	24.8% [7.83 to 44.1%]
Partial hepatectomies	23.0% [12.6 to 34.4%]
Lobectomy ± partial hepatectomy	26.0% [− 23.3 to 105.1%]

### Characteristics associated with MIS vs. open resection approach

In multivariable analysis, patients were less likely to undergo MIS resection if they resided in a county with less than 250,000 people (vs. > 250,000, odds ratio [OR], 95% confidence interval [CI], 0.82 [0.73–0.93]), had a secondary malignancy (vs. primary malignancy, OR [95% CI], 0.61 [0.53–0.69]), had a neuroendocrine tumor (vs. primary malignancy, OR [95% CI], 0.48 [0.29–0.80]), had multiple indications (vs. primary malignancy OR [95% CI], 0.50 [0.37–0.67]), or had additional Elixhauser comorbidities (OR [95% CI], 0.89 [0.86–0.91] for each additional comorbidity) (Table 3). Patients were more likely to undergo a MIS resection if they were older than the median age of the study population (OR [95% CI], 1.15 [1.02–1.29]), had a benign indication (vs. primary malignancy, OR [95% CI], 2.90 [2.49–3.38]), or were undergoing a partial hepatectomy (vs. lobectomy, OR [95% CI], 2.62 [2.24–3.07]) (Table 3).

**Table 3:** Adjusted odds ratio estimates for minimally invasive vs. open resection

Variable	Adjusted odds ratio (95% confidence interval)
Age	
Below median	Reference
Above or equal to median	**1.15 (1.02–1.29)**
Sex	
Male	Reference
Female	1.00 (0.91–1.10)
Race and ethnicity	
White	Reference
Black	1.08 (0.93–1.26)
Hispanic	1.06 (0.90–1.24)
Asian or Pacific Islander	0.95 (0.76–1.19)
Native American	1.23 (0.63–2.38)
Other	1.20 (0.95–1.51)
Insurance status	
Private	Reference
Medicare	1.03 (0.91–1.17)
Medicaid	0.97 (0.82–1.15)
Self-pay	0.80 (0.54–1.18)
No charge	0.46 (0.17–1.24)
Other	1.33 (1.00–1.78)
Rurality	
> = 250,000	Reference
< 250,000	**0.82 (0.73–0.93)**
Income by zip code	
Below median	Reference
Above median	1.03 (0.93–1.15)
Indication	
Primary malignant	Reference
Secondary malignant	**0.61 (0.53–0.69)**
Neuroendocrine tumor	**0.48 (0.29–0.80)**
Benign	**2.90 (2.49–3.38)**
Multiple	**0.50 (0.37–0.67)**
Other	1.00 (0.86–1.17)
Surgery type	
Lobectomy	Reference
Lobectomy and partial hepatectomy	**0.37 (0.20–0.70)**
Partial hepatectomy	**2.62 (2.24–3.07)**
Number of elixhauser comorbidities	**0.89 (0.86–0.91)**
Hospital location/teaching status	
Rural	Reference
Urban non-teaching	0.90 (0.52–1.55)
Urban teaching	0.70 (0.43–1.15)
Hospital region	
Northeast	Reference
Midwest	0.85 (0.69–1.04)
South	0.98 (0.82–1.18)
West	1.00 (0.83–1.22)

Bolded values indicate statistical significance (*p* < 0.05)

### Robotic-assisted vs. laparoscopic liver resections

In the subgroup analysis restricted to MIS recipients, baseline characteristics of patients who received robotic-assisted versus laparoscopic liver resection are presented in Supplemental Table 1. In multivariable analyses, patients with Medicaid (vs. private insurance, OR [95% CI], 0.71 [0.51–0.98]) and patients with self-pay (vs. private insurance, OR [95% CI], 0.32 [0.12–0.85]) were less likely to undergo a robotic-assisted compared to a laparoscopic liver resection (Supplemental Table 2). Indication was not associated with resection approach. However, patients undergoing a partial hepatectomy (vs. lobectomy, OR [95% CI], 0.70 [0.52–0.95]) were less likely to have a robotic-assisted compared to a laparoscopic liver resection. Hospital characteristics were also not associated with resection approach (Supplemental Table 2).

### Post-operative outcomes of MIS vs. open resections

Table 4 presents the distribution of post-operative complications, overall and by resection approach. Overall, the most common types of complications included end organ dysfunction (26.0%), transfusion (7.7%), and infectious complication (5.1%). The mean length of stay was 6.2 days and 1.3% of the population died in the hospital. After accounting for demographic, clinical, and hospital characteristics, a MIS compared to an open approach was associated with reduced odds of infectious complications (OR [95% CI], 0.39 [0.3–0.51]), wound complications (OR [95% CI], 0.26 [0.15–0.47]), end organ dysfunction (OR [95% CI], 0.52 [0.46–0.58]), thromboembolic complications (OR [95% CI], 0.51 [0.32–0.81]), accidental laceration or puncture (OR [95% CI], 0.32 [0.14–0.73]), transfusion (OR [95% CI], 0.31 [0.24–0.40]) and in-hospital mortality (OR [95% CI], 0.46 [0.26–0.83]) (Table 5).

**Table 4:** Distribution of complications in open vs. minimally invasive resections

	Overall	Open	Minimally invasive
	*N* = 63,280*	*N* = 49,045	*N* = 14,235
Infectious complication	3250 (5.1)	2955 (6.0)	295 (2.1)
Wound complication	1030 (1.6)	970 (2.0)	60 (0.4)
End organ dysfunction	16,475 (26.0)	14,290 (29.1)	2185 (15.3)
Thromboembolic complication	1,015 (1.6)	910 (1.9)	105 (0.7)
Accidental laceration or puncture	435 (0.7)	400 (0.8)	35 (0.2)
Transfusion	4865 (7.7)	4475 (9.1)	390 (2.7)
Other complication	235 (0.4)	200 (0.4)	35 (0.2)
In-hospital mortality	805 (1.3)	735 (1.5)	70 (0.5)
Length of stay, mean (standard deviation)	6.2 (6.5)	7.1 (6.9)	3.4 (3.7)

**Table 5:** Odds ratio estimates for complications in minimally invasive vs. open resections

Complication type	Adjusted odds ratio (95% confidence interval)
Infectious complications	0.39 (0.30–0.51)
Wound complications	0.26 (0.15–0.47)
End organ dysfunction	0.52 (0.46–0.58)
Thromboembolic complications	0.51 (0.32–0.81)
Accidental laceration or puncture	0.32 (0.14–0.73)
Transfusion	0.31 (0.24–0.40)
Other complications	0.66 (0.28–1.53)
In-hospital mortality	0.46 (0.26–0.83)

*Each multivariable logistic regression model accounted for age, sex, race/ethnicity, insurance status, rurality, income by zip code, indication, surgery type, Number of Elixhauser Comorbidities, hospital location, hospital teaching status and hospital region

In subgroup analyses within the MIS resection group, Supplemental Table 3 presents the distribution of post-operative complications, overall and by laparoscopic and robotic-assisted approaches. The most common types of complications included end organ dysfunction (15.3%), transfusion (2.7%), and infectious complication (2.1%). The mean length of stay was 3.4 days and 0.5% of the population died in the hospital. After accounting for demographic, clinical, and hospital characteristics, the odds of post-operative complications were similar for most individual outcomes for robotic-assisted vs. laparoscopic resection. The exception was that the odds of transfusion was higher among robotic-assisted versus laparoscopic liver resections (OR [95% CI], 1.71 [1.02–2.86]), however the precision of the estimate was wide. No difference in the odds of in-hospital mortality by MIS approach was observed (Supplemental Table 4).

## Discussion

From 2016 to 2020, there was a modest increase in MIS resections driven by an increase in the proportion of robotic-assisted liver resections and despite a reduction in the proportion of laparoscopic liver resection. The decline of laparoscopic and increase in robotic-assisted liver resections was more pronounced with partial hepatectomies compared to lobectomies. Selection of a MIS approach vs. an open approach was independently associated with patient rurality, indication, and surgery type. A robotic-assisted vs. laparoscopic resection approach did not appear to be influenced by indication but was instead associated with insurance type and surgery type. Furthermore, this study corroborated existing evidence in favor of improved post-operative outcomes associated with MIS vs. open liver resection. [4, 6, 16]. When adjusting for demographic, clinical, and hospital characteristics, patients undergoing MIS resections had decreased post-operative complications and reduced in-hospital mortality compared to patients undergoing open resections.

National utilization of MIS liver resections was previously examined in the context of the International Louisville Statement [9]. Based on NIS and National Surgical Quality Improvement Program (NSQIP) data, there was a progressive increase in laparoscopic liver resection following the Louisville statement during the years from 2009 to 2012. However, in 2012, the laparoscopic approach only made up roughly 6% of all liver resections. An additional examination of 2012 NIS data showed that when controlling for patient and hospital characteristics, laparoscopic liver resection was associated with 40% lower odds of developing postoperative complications compared to open for malignancy [8]. 

Our study provides an update to these two prior studies and expands the literature by characterizing robotic-assisted in addition to laparoscopic liver resections. In 2016, MIS liver resections made up 21.3% of all liver resections, which is notably higher than the approximately 6% in 2012. While this increase may be affected by the shift from ICD-9 to ICD-10 codes that occurred in 2015, the increase in MIS liver resections and proportion of total resections that are MIS are consistent with single institution and survey-based studies [17, 18]. Additionally, this study shows a 44% to 74% reduction in odds of post operative complications and a 54% reduction in odds of in-hospital mortality**.** This corroborates the reduction in post-operative complications reported in other studies [4, 8, 16]. Despite these improved short-term outcomes, open resections continue to comprise over 75% of all liver resections.

There are likely a number of factors that contribute to decreased MIS utilization nationally. First, concerns about long-term oncologic outcomes likely continue to be influential despite evidence showing non-inferiority [19]. The difference in distribution of indication between MIS and open resections suggests that surgeons are more willing to use MIS approaches for benign indications rather than malignant indications. High quality evidence, in primary hepatobiliary malignancies in addition to colorectal liver metastases may help to increase utilization of MIS approaches for malignancies. Second, surgeon preference and experience are important factors to consider and this is highlighted by the dynamic between laparoscopic and robotic-assisted liver resection. The expansion of robotic-assisted resections over the study period appears to be mostly at the expense of laparoscopic rather than open. Due to the earlier availability and adoption of laparoscopic liver resections, utilization of a robotic-assisted approaches generally follows laparoscopic experience [17, 20]. The growth in robotic-assisted and decline of laparoscopic liver resections is consistent with the hypothesis that surgeons who utilize laparoscopy have shifted part of their MIS practice to robotic-assisted rather than surgeons who mostly perform open expanding to robotic-assisted. Surgeons with significant open experience and with limited MIS experience may be less likely to adopt MIS approaches, even with evidence supporting improved post-operative outcomes. Continuing to report on the post-operative benefits of MIS liver resections, along with MIS training designed for established surgeons could help increase MIS utilization in this surgeon population.

Finally, even amongst surgeons with significant MIS training experience, MIS resections are likely limited to more minor resections rather than major anatomic resections [21]. Large anatomic resections are likely only being performed minimally invasively at a handful of centers. The steeper learning curve associated with these resections limits the number of resections that can be performed minimally invasively, even by the average surgeon with significant MIS experience.

Within MIS resections, the decision between a laparoscopic and robotic-assisted approach is complex, with both clinical factors and access playing significant roles. From a clinical perspective, patients undergoing a lobectomy, compared to a partial hepatectomy, were more likely to have a robotic-assisted approach rather than a laparoscopic approach. This is consistent with a single-center experience indicating that more complex resections were performed robotically compared to laparoscopically [17]. From an access perspective, patients with Medicaid or self-pay, compared to private insurance, were less likely to undergo a robotic-assisted vs. a laparoscopic resection. At this time, there is no definitive evidence to choose between laparoscopic and robotic-assisted based on outcomes. Complications, length of stay, and in-hospital mortality were not significantly different in this study population. A meta-analysis on this subject showed similar rates of perioperative morbidity, rate of conversion to open, and total length of stay between the two groups [22]. While that study also showed similar rates of positive resection margins, long-term oncologic outcomes have not been as robustly reported.

Results of this study should be interpreted in the context of the inherent limitations of administrative databases and specifically the NIS [23, 24]. First, the results may be subject to residual confounding due to the nonrandomized nature of the study design. Our estimates of the association between resection approach and post-operative outcomes accounted for demographic, clinical, and hospital characteristics, however the NIS does not include information on potential confounders such as tumor characteristics (e.g., tumor size, location, pathology, and invasion) or complexity of the operation. Second, misclassification of post-operative complications is possible; however, ICD-10 codes that have been verified for accurate estimation of complications in administrative data were used [13]. Third, suboptimal classification of confounding variables, specifically indication and surgery type, is also possible. The specific indication for surgery may not be accurately captured in every patient’s ICD-10 codes, leading to a classification of “other.” Additionally, the ICD-10 procedure codes for liver resections are limited and may not adequately capture the complexity and specifics of every resection. All patients with liver resections were included rather than limiting by indication as this allowed us to broadly characterize the use of MIS approaches in a nationally representative sample, even capturing vulnerable populations including Medicaid and uninsured patients. However, this does lead to a more heterogenous sample with the possibility for misclassification of indication. The cross-sectional nature of the NIS database also prevents longitudinal analysis of long-term outcomes, including quality of resection margins, disease-free survival, and overall survival. Furthermore, we did not examine the rate of conversion from MIS to open procedures. This is an important dynamic to address moving forward. Decreased rates of conversion over time could provide insight into increased proficiency with MIS approaches, however this could be confounded by selection of more complicated resections to attempt MIS. With the validation and increased utilization of conversion related ICD-10 codes, these dynamics could be elucidated in subsequent studies.

Finally, while the increase in MIS liver resections compared to prior studies likely represents a true expansion of laparoscopic liver resections, along with the addition of robotic-assisted liver resection, it is important to acknowledge the shift from ICD-9 codes to ICD-10 codes that occurred in 2015 [8, 9]. For liver resections prior to 2016, ICD-9 codes were used to classify resections as either “partial hepatectomy” or “lobectomy.” However, ICD-10 codes shifted to “excision of liver” or “resection of liver”, with codes having right and left specifiers. Additionally, coding of laparoscopic resections with ICD-9 codes required the addition of a specific laparoscopic code, while the ICD-10 codes have “open approach”, or “percutaneous endoscopic approach” attached to each code. While the previous studies did not consider robotic-assisted liver resections, both ICD-9 and ICD-10 codes require an additional code for specification of a robotic assisted procedure.

## Conclusion

Using a nationally representative sample of liver resections over five years (2016–2020), the use of a MIS approach increased slightly from 2016 to 2020 driven by an increase in robotic-assisted resections and despite a reduction in laparoscopic resections. Even though MIS approaches were associated with reduced post-operative complications and in-hospital mortality, open resections continued to predominate. MIS approaches continue to be underutilized, particularly in patients with benign indications. The expansion of MIS approaches may be slowed by the switch from laparoscopic to robotic-assisted resections. However, robotic-assisted resections allow for more complex resections to be completed with a MIS approach and will likely expand the overall MIS share in the long-term. Training that focuses on proficiency in MIS and open approaches will be crucial for optimization of outcomes for patients undergoing liver resections.

## Supplementary Information

Below is the link to the electronic supplementary material.

Supplementary file1 (DOCX 28 KB)

Supplementary file2 (XLS 15 KB)
