# Supplementary material for: National utilization of minimally invasive and open liver resections
Source: Surg Endosc. 2026 Apr 20;40(7):5780–9. doi: 10.1007/s00464-026-12678-9 (PMC13369177; doi:10.1007/s00464-026-12678-9)
Supplement: Supplementary file 1 — Supplementary file1 (DOCX 28 KB) [file 464_2026_12678_MOESM1_ESM.docx]

**Supplementary Tables**

***Supplemental Table 1. Demographic, Clinical, and Hospital Characteristics of Patients in MIS Population by Laparoscopic vs. Robotic-Assisted Approach***

|  | **MIS** | **Laparoscopic** | **Robotic-Assisted** | **Standardized Mean Difference** |
| --- | --- | --- | --- | --- |
|  | **N = 14,235*** | **N = 10,805** | **N = 3,430** |  |
| **Patient Demographics** |  |  |  |  |
| Age, mean (standard deviation) | 59.4 (14.2) | 59.4 (14.3) | 59.6 (13.9) | 0.02 |
| Sex |  |  |  | 0.12 |
| Male | 5,970 (41.9) | 4,375 (40.5) | 1,595 (46.5) |  |
| Female | 8,265 (58.1) | 6,430 (59.5) | 1,835 (53.5) |  |
| Race and Ethnicity |  |  |  | 0.08 |
| White | 9,635 (67.7) | 7,310 (67.7) | 2,325 (67.8) |  |
| Black | 1,540 (10.8) | 1,160 (10.7) | 380 (11.1) |  |
| Hispanic | 1,515 (10.6) | 1.115 (10.3) | 400 (11.7) |  |
| Asian or Pacific Islander | 845 (5.9) | 660 (6.1) | 185 (5.4) |  |
| Native American | 55 (0.4) | 40 (0.4) | 15 (0.4) |  |
| Other | 645 (4.5) | 520 (4.8) | 125 (3.6) |  |
| Insurance |  |  |  | 0.15 |
| Private Insurance | 6,700 (47.2) | 4,995 (46.3) | 1,705 (50.0) |  |
| Medicare | 5,480 (38.6) | 4,160 (38.5) | 1,320 (38.7) |  |
| Medicaid | 1,325 (9.3) | 1,070 (9.9) | 255 (7.5) |  |
| Self-Pay | 235 (1.7) | 210 (1.9) | 25 (0.7) |  |
| No Charge | 25 (0.2) | 20 (0.2) | *** |  |
| Other | 440 (3.1) | 340 (3.1) | 100 (2.9) |  |
| Rurality |  |  |  | < 0.01 |
| Greater than or equal to 250,000 | 11,600 (81.5) | 8,805 (81.5) | 2,795 (81.5) |  |
| Less than 250,000 | 2,635 (18.5) | 2,000 (18.5) | 635 (18.5) |  |
| Income by Zip Code |  |  |  | 0.04 |
| Above Median | 7,960 (55.9) | 6,095 (56.4) | 1,865 (54.4) |  |
| Below Median | 6,275 (44.1) | 4,710 (43.6) | 1,565 (45.6) |  |
| **Patient Clinical Characteristics** |  |  |  |  |
| Indication |  |  |  | 0.16 |
| Primary Malignancy | 3,260 (22.9) | 2,460 (22.8) | 800 (23.3) |  |
| Secondary Malignancy | 4,190 (29.4) | 3,020 (28.0) | 1,170 (34.1) |  |
| Neuroendocrine Tumor | 110 (0.8) | 85 (0.8) | 25 (0.7) |  |
| Benign | 3,410 (24.0) | 2,665 (24.7) | 745 (21.7) |  |
| Multiple | 260 (1.8) | 185 (1.7) | 75 (2.2) |  |
| Other | 3,005 (21.1) | 2,390 (22.1) | 615 (17.9) |  |
| Surgery Type |  |  |  | 0.11 |
| Lobectomy | 1,225 (8.6) | 850 (7.9) | 375 (10.9) |  |
| Lobectomy and Partial Hepatectomy | 60 (0.4) | 40 (0.4) | 20 (0.6) |  |
| Partial hepatectomy | 12,950 (91.0) | 9,915 (91.8) | 3,035 (88.5) |  |
| Number of Elixhauser Comorbidities,  mean (standard deviation) | 2.68 (1.69) | 2.69 (1.70) | 2.67 (1.64) | < 0.01 |
| **Hospital Characteristics** |  |  |  |  |
| Hospital Bed Size |  |  |  | 0.12 |
| Small | 1,040 (7.3) | 865 (8.0) | 175 (5.1) |  |
| Medium | 2,985 (21.0) | 2,290 (21.2) | 695 (20.3) |  |
| Large | 10,210 (71.7) | 7,650 (70.8) | 2,560 (74.6) |  |
| Hospital Location/Teaching Status |  |  |  | 0.03 |
| Rural | 150 (1.1) | 115 (1.1) | 35 (1.0) |  |
| Urban Non-Teaching | 945 (6.6) | 695 (6.4) | 250 (7.3) |  |
| Urban Teaching | 13,140 (92.3) | 9,995 (92.5) | 3,145 (91.7) |  |
| Hospital Region |  |  |  | 0.11 |
| Northeast | 3,510 (24.7) | 2,760 (25.5) | 750 (21.9) |  |
| Midwest | 2,780 (19.5) | 2,110 (19.5) | 670 (19.5) |  |
| South | 4,830 (33.9) | 3,545 (32.8) | 1,285 (37.5) |  |
| West | 3,115 (21.9) | 2,390 (22.1) | 725 (21.1) |  |

*Percentages presented in parentheses, unless indicated otherwise

****Not reportable due to small sample size restrictions of HCUP data*

*Supplemental Table 2. Adjusted Odds Ratio Estimates for Robotic-Assisted vs. Laparoscopic Resection*

| **Variable** | **Adjusted Odds Ratio (95% Confidence Interval)** |
| --- | --- |
| Age |  |
| Below Median | Reference |
| Above or Equal to Median | 1.04 (0.83-1.30) |
| Sex |  |
| Male | Reference |
| Female | 0.83 (0.68-1.01) |
| Race and Ethnicity |  |
| White | Reference |
| Black | 1.04 (0.78-1.40) |
| Hispanic | 1.18 (0.88-1.58) |
| Asian or Pacific Islander | 0.95 (0.63-1.43) |
| Native American | 1.39 (0.33-5.80) |
| Other | 0.77 (0.49-1.19) |
| Insurance Status |  |
| Private | Reference |
| Medicare | 0.89 (0.72-1.12) |
| **Medicaid** | **0.71 (0.51-0.98)** |
| **Self-Pay** | **0.32 (0.12-0.85)** |
| No Charge | 0.54 (0.06-5.15) |
| Other | 0.83 (0.47-1.47) |
| Rurality |  |
| >= 250,000 | Reference |
| < 250,000 | 0.94 (0.71-1.24) |
| Income by Zip Code |  |
| Below Median | Reference |
| Above Median | 0.92 (0.76-1.12) |
| Indication |  |
| Primary Malignant | Reference |
| Secondary Malignant | 1.18 (0.91-1.53) |
| Neuroendocrine Tumor | 0.90 (0.34-2.42) |
| Benign | 0.91 (0.68-1.22) |
| Multiple | 1.21 (0.64-2.28) |
| Other | 0.80 (0.60-1.07) |
| Surgery Type |  |
| Lobectomy | Reference |
| Lobectomy and Partial Hepatectomy | 1.01 (0.27-3.79) |
| **Partial hepatectomy** | **0.70 (0.52-0.95)** |
| Number of Elixhauser Comorbidities | 0.98 (0.92-1.04) |
| Hospital Location/Teaching Status |  |
| Rural | Reference |
| Urban Non-Teaching | 1.99 (0.63-6.24) |
| Urban Teaching | 1.64 (0.57-4.75) |
| Hospital Region |  |
| Northeast | Reference |
| Midwest | 1.14 (0.78-1.68) |
| South | 1.25 (0.91-1.72) |
| West | 1.01 (0.71-1.44) |

*Supplemental Table 3. Distribution of Complications in Laparoscopic vs. Robotic-Assisted Resections*

|  | **MIS** | **Laparoscopic** | **Robotic-Assisted** |
| --- | --- | --- | --- |
|  | **N = 14,235*** | **N = 10,805** | **N = 3,430** |
| Infectious Complication | 295 (2.1) | 205 (1.9) | 90 (2.6) |
| Wound Complication | 60 (0.4) | 55 (0.5) | *** |
| End Organ Dysfunction | 2,185 (15.3) | 1,650 (15.3) | 535 (15.6) |
| Thromboembolic Complication | 105 (0.7) | 95 (0.9) | *** |
| Accidental Laceration or Puncture | 35 (0.2) | 25 (0.2) | *** |
| Transfusion | 390 (2.7) | 250 (2.3) | 140 (4.1) |
| Other Complication | 35 (0.2) | 15 (0.1) | 20 (0.6) |
| In-Hospital Mortality | 70 (0.5) | 50 (0.5) | 20 (0.6) |
| Length of Stay, mean (standard deviation) | 3.4 (3.7) | 3.4 (3.6) | 3.5 (4.1) |

****Not reportable due to small sample size restrictions of HCUP data*

*Supplemental Table 4. Odds Ratio Estimates for Complications in Robotic-Assisted vs. Laparoscopic Resections*

| **Complication Type** | **Adjusted Odds Ratio (95% Confidence Interval)** |
| --- | --- |
| Infectious Complications | 1.47 (0.79-2.72) |
| Wound Complications | 0.28 (0.03-2.30) |
| End Organ Dysfunction | 0.99 (0.76-1.29) |
| Thromboembolic Complications | 0.28 (0.05-1.54) |
| Accidental Laceration or Puncture | 1.52 (0.21-11.19) |
| Transfusion | 1.71 (1.02-2.86) |
| Other Complications | 4.04 (0.56-29.24) |
| In-Hospital Mortality | 1.03 (0.28-3.82) |

**The multivariable logistic regression model accounted for age, sex, race/ethnicity, insurance status, rurality, income by zip code, indication, surgery type, Number of Elixhauser Comorbidities, and hospital characteristics*
